# Co-Occurrence Patterns of *Ustilago nuda* and *Pyrenophora graminea* and Fungicide Contribution to Yield Gain in Barley under Fluctuating Climatic Conditions in Serbia

**DOI:** 10.3390/jof8050542

**Published:** 2022-05-23

**Authors:** Radivoje Jevtić, Vesna Župunski, Mirjana Lalošević, Ljiljana Brbaklić, Branka Orbović

**Affiliations:** Institute of Field and Vegetable Crops, 21000 Novi Sad, Serbia; vesna.zupunski@ifvcns.ns.ac.rs (V.Ž.); mirjana.lalosevic@ifvcns.ns.ac.rs (M.L.); ljiljana.brbaklic@ifvcns.ns.ac.rs (L.B.); branka.orbovic@ifvcns.ns.ac.rs (B.O.)

**Keywords:** *Hordeum vulgare* L., *Ustilago nuda*, *Pyrenophora graminea*, fungicide efficacy, yield

## Abstract

The utilization of production systems with reduced chemical input renewed the interest in *Ustilago nuda* and *Pyrenophora graminea*. The investigations of seed fungicide treatments are more related to their efficacy than to their contribution to yield gain. The data were collected from research and development trials on fungicide efficacy against *U. nuda* and *P. graminea* conducted from 2014 to 2020 in Serbia. Partial least squares, multiple stepwise regression and best subset regression were used for statistical modeling. The total number of plants infected with *U. nuda* and *P. graminea* per plot differed significantly in the seven-year period. Shifts in the predominance of one pathogen over the other were also shown. Temperature, total rainfall and relative humidity in flowering time (*p* < 0.001) influenced the occurrence of both pathogens. The strongest impact on yield loss was observed for temperature in the phenological phases of leaf development (*p* = 0.014), temperature in flowering time (*p* < 0.001) and total number of plants infected with *U. nuda* and *P. graminea* per plot (*p* < 0.001). Our results indicated that regression models consisting of both biotic and abiotic factors were more precise in estimating regression coefficients. Neither fungicidal treatment had a stable contribution to yield gain in the seven-year period.

## 1. Introduction

Cultivated barley (*Hordeum vulgare* ssp. *vulgare* L.) is an economically important temperate cereal. It is well adapted to diverse environmental stresses, including low rainfall and cold winter temperatures [1]. Barley is primarily grown as feed grain and grain for malting, but its importance in human consumption is also growing [2]. According to USDA Foreign Agricultural Services [3], barley is a secondary grain crop in Serbia. The total barley production area has been increasing since 2013, and in 2020, it took the area of 101,483 ha. Total barley consumption in Serbia takes half for animal feed and half for the brewery industry [3].

The seed-borne fungal pathogens *Ustilago nuda* (Jensen) Rostrup and *Pyrenophora graminea* Ito and Kuribayashi are causal agents of loose smut and barley stripe, respectively [4,5]. These are economically important pathogens and can be found at the same time in barley fields. Infection with *U. nuda* occurs during grain formation, when the fungus penetrates the endosperm. Infected seeds give rise to systemically infected plants. The symptoms are visible as “smutted” grain heads, with masses of black or brown spores that completely replace the grain head. The black spores are then transmitted by the wind to healthy ears [6]. *P. graminea* is a monocyclic pathogen that infects ears by wind dispersion after conidia release from infected leaves. Symptoms of barley stripe are visible on leaves as yellow and brown-colored longitudinal stripes. The fungus survives in kernels as mycelium on the pericarp and spreads from the seed into the seedling through coleorhiza [7]. The heading of plants affected with *P. graminea* is difficult or cannot take place [8].

Yield losses attributed to loose smut in conventional barley production are of no major concern due to the use of certified seed and fungicidal seed treatments. As a result, little research on the biology, etiology and epidemiology of *U. nuda* has been done since the 1960s, when systemic fungicidal seed treatments became available [4]. However, with the development of fungicide resistance as well as the increasing importance of production systems with reduced chemical input, interest in seed-borne diseases is renewed [4]. Lamichhane et al. [9] indicated that a reduction in fungicide seed treatment in organic production can lead to the reemergence of smuts in cereals. *P. graminea* causes poor heading, and the grain is not fully filled. Yield loss in susceptible varieties could exceed 70% [10] and is attributed to a reduction in the number of spikes, grain size and tillers [10,11]. *P. graminea* is well controlled in conventional production; however, in organic production or when an untreated farmer-saved seed is sown, the disease can reappear [6].

Currently, significant concerns have been raised by the scientific community regarding the impacts of climate change on the future yield potentials of cereals. Climatic factors affect the beginning and duration of plant phenological stages as well as the life cycle and distribution of pathogens. Although all of these factors have a direct impact on yield, yield components and plant–pathogen interactions [10,12,13,14], little is known about the combined effect of abiotic and biotic factors on final yield losses [15,16]. In addition, the efficacy of systemic fungicide seed treatments in controlling barley pathogens is usually reported for one growing season or in investigations concerning fungicide resistance. However, their contribution to yield gain under fluctuating climatic conditions over a longer time period is rarely reported.

Lamichhane [17] indicated that re-evaluation of the routine-based planting of pesticide seed treatment is needed from economic, environmental, and social points of view, although planting pesticide-treated seeds was proven to be an important practice for the control of seed-borne and soil-borne diseases. You et al. [18] also indicated that the effectiveness of fungicides against individual soil-borne pathogens can differ from their effectiveness against soil-borne pathogen complexes. Since there is little knowledge on the patterns of joined occurrence of *U. nuda* and *P. graminea* in barley production areas and their effect on barley yield loss under fluctuating climatic conditions, the aim of this study was to investigate how joined occurrence of *U. nuda* and *P. graminea* affects barley yield, and whether fluctuating climatic conditions affect control of these pathogens. Consequently, the objectives of this study were to examine (1) the co-occurrence patterns of *U. nuda* and *P. graminea* in naturally infected seeds and (2) the combined effect of multiple-seed borne diseases and climatic conditions on yield loss of susceptible barley varieties. We also investigated the contribution of seed fungicide treatment to the yield gain of susceptible barley varieties under fluctuating climatic conditions and diverse pressures of *U. nuda* and *P. graminea* over a seven-year period. The data were collected from 2014 to 2020 and analyzed in terms of agroecological conditions characteristic of Serbia.

The majority of the territory of the Republic of Serbia is under a warm temperate—fully humid climate type with warm summers (Cfb type, according to the Köppen−Geiger Climate Classification). From 1961 until the present, a significant increase in temperature change and change in precipitation patterns were observed [19]. Hence, this study provides new insights on factors affecting co-occurrence of *U. nuda* and *P. graminea* and their contribution to barley yield achievements. It also reveals that combining seed-borne pathogen infection with abiotic predictor variables into the same regression model would give more reliable conclusions on factors affecting yield loss. Neither fungicidal treatment had a stable contribution to yield gain in the seven-year period, which was affected with extreme fluctuations in climatic factors at the time of flowering, overall pathogen pressure and predominance of one pathogen over the other.

## 2. Materials and Methods

### 2.1. Data Origin

Data for this study were obtained from research and development trials (R&D) on fungicide efficacy against *U. nuda* and *P. graminea***.** Trials were conducted from 2014 to 2020 under the direction of the Institute of Field and Vegetable Crops, Novi Sad, Serbia, at the locality of Rimski šančevi (45°19′4730″ N, 19°50′4400″ E, and altitude 85.7 m), Vojvodina, northern province of Serbia.

### 2.2. Field Trial

Field trials were set up using naturally infected seeds of the susceptible variety Krajišnik. Seeds infected with both *U. nuda* and *P. graminea* were used from the previous growing season and collected every year from untreated plots. Cultivation practice was low-input and included plowing. The soil was a slightly carbonated loamy chernozem. In the history of the trial field, there was no record of seedling disease/damping off. The sowing, flowering and harvesting dates of variety Krajišnik are presented in Table 1.

The field trials included 13 fungicides caring for different types of active ingredients (Table 2) and were arranged in a randomized block design comprising four replicates. The plot size of each replicate was 5 m^2^. The seed treatments against *P. graminea* and *U. nuda* were carried out a few days before sowing using the procedure described by EPPO [20].

### 2.3. Disease and Fungicide Efficacy Assessments

Disease assessments were performed in accordance with the EPPO [20] standard by counting the total number of diseased plants per plot (Figure S1). EPPO [20] also includes emergence testing to ensure validity of the data. Assessments of plants infected with *U. nuda* and *P. graminea* were made at the growth stage 61–69 BBCH (beginning of flowering: first anthers visible—end of flowering: all spikelets have completed flowering, but some dehydrated anthers may remain) [20]. The symptoms of loose smut are visible as “smutted” grain heads, with masses of black or brown spores that completely replace the grain head. Symptoms of barley stripe are visible on leaves as yellow and brown-colored longitudinal stripes. In addition, the heading of plants affected with *P. graminea* is difficult or cannot take place.

Fungicide efficacy was calculated using Abbott’s formula for each pathogen (Equation (1)).

Efficacy (%) = (X − Y)/X × 100(1)

X—Number of plants infected with *U. nuda* or *P. graminea* in the seed-untreated plot;
Y—Number of plants infected with *U. nuda* or *P. graminea* in the seed-treated plot.

### 2.4. Yield

Yield was measured for each plot at 15% water content. Yield loss (%) was determined as the average yield reduction in seed-untreated plots compared with the maximum value of average yield in seed-treated plots after comparison of 13 fungicides (Equation (2)).

Y (%) = ((Y_1_ − Y_2_)/Y_1_) × 100 (2)

Y_1_—Maximum average grain yield in seed-treated plots obtained from four replications;
Y_2_—Average grain yield in seed-untreated plots obtained from four replications.

Yield gain (%) was calculated for each fungicide by taking into account average yield in seed-untreated plots and average yield achievement in seed-treated plots (Equation (3)).

Y (%) = ((Y_1_ − Y_2_)/Y_2_) × 100 (3)

### 2.5. Statistical Methods

Multiple stepwise regression was used to investigate the most influencing factors on the occurrence of *U. nuda* and *P. graminea* in 2014–2020. Predictor variables for the identification of climatic factors influencing the occurrence of *P. graminea* and *U. nuda* in seed-untreated plots were those that were previously reported to be the most important for the initiation of infection and disease development and included climatic factors at the time of flowering (averages of temperature, total rainfall and humidity in the 10-day period at the time of flowering in the previous growing season when infection occurred); averages of temperature, total rainfall and humidity in the 10-day period at the time of sowing; and monthly averages of temperatures, relative humidity and total rainfall taken at the time of seed germination and leaf development in November at the experimental site (http://www.hidmet.gov.rs/, accessed on 1 September 2021). The weather station of the Republic Hydrometeorological Institute of Serbia is located on Rimski šančevi, the same as the experimental site. Tukey pairwise comparison was used to identify whether the total number of plants infected with *U. nuda* and *P. graminea* per plot differed among growing seasons.

The effects of the year and fungicidal treatments on the yield of variety Krajišnik were analyzed using ANOVA (general linear model) with year as a fixed factor. Three types of regression models were used to investigate influencing factors on yield loss due to multicollinearity of data. These were partial least squares (PLS), multiple stepwise regression and best subset regression.

PLS regression was performed to extract a set of the most influential climatic factors on yield loss in the seed-untreated plots. Monthly averages of temperatures, relative humidity and total rainfall taken from November to June for each growing season (http://www.hidmet.gov.rs/, accessed on 1 September 2021) were taken into consideration. The technique applied in PLS analysis is similar to principal component analysis because it gives the option of leave-one-out cross-validation to maximize the model’s predictive ability. After PLS analysis, the combined effect of abiotic and biotic factors on yield loss was analyzed using both stepwise and best subset regression. Climatic factors selected in PLS regression (abiotic independent variables) and joined occurrence of *U. nuda* and *P. graminea* (biotic independent variable) were further used as predictors in both best subsets and stepwise regression analysis.

Best subset regression was undertaken to compare regression models consisting of both abiotic and biotic predictors and to examine how much variation in yield loss will be explained by the maximum R^2^ criterion. Mallows’ Cp was used to make comparisons between multiple regression models. A small value of Mallows’ Cp indicated that the model is relatively unbiased in estimating the true regression coefficients. Stepwise regression was performed to confirm the results obtained by best subset regression. While performing stepwise regression, alpha to enter and alpha to remove the influencing factors was set by default to be 0.15, since it was reported that a level such as 0.05 can fail in identifying variables known to be important [21]. Regression models were followed with the coefficient of determination (R^2^) showing the percentage of variation in the response that was explained by the model.

Spearman’s coefficient of correlation was used to identify the correlation between yield loss and the combined occurrence of *U. nuda* and *P. graminea*. The relationship between yield gain in seed-treated plots and the pathogen pressure of *U. nuda* and *P. graminea* was analyzed using polynomial regression. The pathogen pressure of *U. nuda* and *P. graminea* was expressed as the total number of plants infected with *U. nuda* and *P. graminea* per seed-untreated plot (5 m^2^) in each growing season. Minitab 17 (trial version) was used for the entire analysis.

## 3. Results

The average yield of the variety Krajišnik in seed-treated and seed-untreated plots differed significantly among the years (*p* < 0.001). The average yield ranged from 3.5 to 11.8 t/ha in the seed-treated plots and 3.4 to 10.6 t/ha in the seed-untreated plots. The yields in the seed-treated plots were significantly different (*p* < 0.001) from those in the seed-untreated plots. A general linear model (ANOVA) conducted with fungicide treatment and year as predictor variables explained the variability of the yield with a coefficient of determination (R^2^) of 89.5%. The yield was significantly influenced by both year (*p* < 0.001) and fungicide treatment (*p* < 0.001).

The average yield loss of the variety Krajišnik in the seed-untreated plots in the seven-year period was 16.85% and ranged from 6.7% to 29.5%. From 2016 to 2019, the yield loss ranged from 17.9 to 29.5%, which exceeded the seven-year period average.

### 3.1. Co-Occurrence Patterns of U. nuda and P. graminea in Agro-Ecological Conditions in Serbia

**Figure 1:**
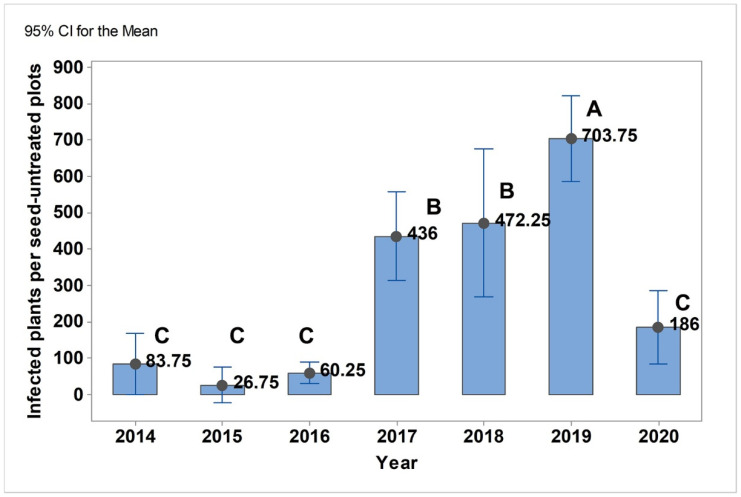
Joined occurrence of *U. nuda* and *P. graminea* in seed-untreated plots in 2014–2020. Means that do not share a letter are significantly different.

*U. nuda* and *P. graminea* had different co-occurrence patterns during the seven-year period (Figure S2). *U. nuda* predominated over *P. graminea* from 2014–2017 and reached a maximum value of 314 infected plants per seed-untreated plot in 2017. The occurrence of *P. graminea* was less than 122 infected plants per seed-untreated plot in the same time period. In 2018, the occurrence of *U. nuda* also exceeded the seven-year average, but *P. graminea* predominated, with 258 infected plants per seed-untreated plot (Figure S2). From 2018 to 2020, *P. graminea* was the predominant pathogen and reached a maximum of 688 infected plants per seed-untreated plot in 2019 (Figure S2). Tukey’s test indicated that the combined occurrence of *U. nuda* and *P. graminea* differed significantly in the seven-year period (*p* < 0.001) (Figure 1).

The most influential climatic factors on the occurrence of *U. nuda* and *P. graminea* in the seed-untreated plots were examined using stepwise regression. Temperature, total rainfall and humidity in the 10-day period at the time of flowering (in the previous growing season when infection occurred) had a significant influence on the occurrence of both pathogens (Table 3). In the years that preceded the highest occurrence of *U. nuda* (2017 and 2018), the temperatures at the time of flowering of 14.3 °C and 14.7 °C and total rainfall of 27.1 mm and 28.7 mm were near the seven-year average (Table S1). The relative humidity at the time of flowering did not exceed 79% in the seven-year period (Table S1). The maximum infection with *P. graminea* occurred in 2019, when the temperature at flowering time in the previous growing season reached a maximum value in the seven-year period (20.5 °C) and exceeded the seven-year period average (16.9 °C).

Temperature in November influenced the occurrence of only *U. nuda* (*p* < 0.001) (Table 3). The maximum occurrence of *U. nuda* was in 2017 and 2018, when the average temperature in November in the seven-year period had the lowest values of 6.3 °C and 7 °C, causing slow plant growth, which additionally promoted *U. nuda* occurrence.

Average temperature and total rainfall in the 10-day period at the time of sowing were distinguished as factors affecting the occurrence of only *P. graminea* (*p* < 0.001) (Table 3). In our study, the average soil temperature at the time of sowing ranged from 8.5 °C (2014) to 15 °C (2013), and when *P. graminea* reached the maximum value, the average temperature at the time of sowing was 14 °C.

### 3.2. Combined Effect of Multiple Seed-Borne Diseases and Climatic Factors on Yield Loss in Seed-Untreated Plots

**Figure 2:**
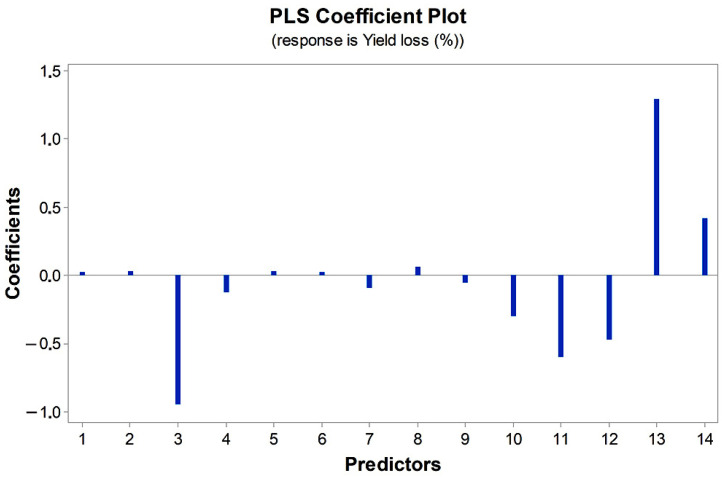
Partial least square coefficient plot of climatic factors influencing yield loss of variety Krajišnik in the period 2014–2020; (1) Total rainfall in November, (2) Total rainfall in December, (3) T in November, (4) T in December, (5) Total rainfall in January, (6) Total rainfall in February, (7) Total rainfall in March, (8) Total rainfall in April, (9) Total rainfall in May, (10) T in January, (11) T in February, (12) T in March, (13) T in April, (14) T in May.

PLS regression was undertaken to reduce the abiotic predictors to a smaller set of uncorrelated components. The most influential abiotic predictors on yield loss selected by PLS were average temperature in November, February and April (Figure 2). These factors had the highest absolute values of coefficients.

Selected predictors of abiotic factors together with the total number of plants infected with *U. nuda* and *P. graminea* per plot were subjected to stepwise and best subset regression analysis. The best subset regression revealed that temperature in November, temperature in April, and total number of plants infected with *U. nuda* and *P. graminea* per seed-untreated plot gave the model with the smallest Mallows’ Cp (4.0) (Table 4). This indicated that this model was relatively unbiased in estimating the true regression coefficients. It also showed that using solely abiotic or biotic predictor variables in regression analysis would give higher Mallows’ Cp in contrast to regression models with combined abiotic and biotic predictor variables. This study indicated that abiotic and biotic factors influencing yield losses of barley should be analyzed together.

Stepwise regression was conducted to confirm the results obtained by best subset regression. Stepwise and best subset regression analyses revealed the same influencing factors on yield loss of variety Krajišnik. Temperature in November (*p* = 0.014), temperature in April (*p* < 0.001) and total number of plants infected with *U. nuda* and *P. graminea* per seed-untreated plot (*p* < 0.001) gave the model with R^2^ (77.7%) and R^2^ (pred) (71.6%).

It should be pointed out that, although the combined occurrence of *U. nuda* and *P. graminea* in 2019 (703 infected plants per seed-untreated plot) was almost twice that in 2017 (436 infected plants per seed-untreated plot), the yield loss (20.6%) was similar to that in 2017 (19.7%). Since *P. graminea* predominated in 2019 (688 plants per seed-untreated plot) and *U. nuda* in 2017 (314 plants per seed-untreated plot), these results indicated different contributions of pathogens to final yield achievements. Moreover, the joint occurrence of *U. nuda* and *P. graminea* in 2018 (472 infected plants per seed-untreated plot) and 2017 (436 infected plants per seed-untreated plot) was not significantly different, but yield loss in 2018 (29.5%) exceeded that in 2017 (19.7%). Although the correlation between yield loss and the total number of plants infected with *U. nuda* and *P. graminea* per seed-untreated plot was highly positive (r = 0.762, *p* < 0.001), regression models indicated that the prediction of barley yield loss should not be based only on the data of the occurrence of loose smut and barley stripe.

### 3.3. The Contribution of Fungicide Treatment to Yield Gain

**Figure 3:**
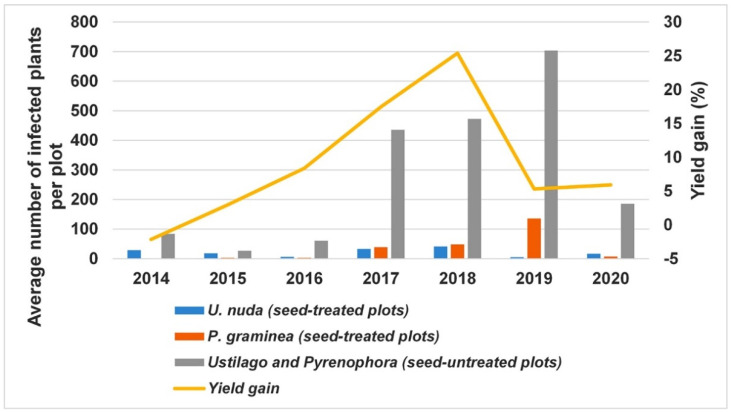
Relationship between *U. nuda* and *P. graminea* occurrence in seed-treated and seed-untreated plots and yield gain of variety Krajišnik in the period 2014–2020.

Yield gain in seed-treated plots did not follow a linear pattern in the seven-year period (Figure 3). The lowest contribution of fungicide treatment to the yield gain (<3%) was observed in 2014 and 2015, when the total number of plants infected with *U. nuda* and *P. graminea* per seed-untreated plot ranged from 26 (2015) to 84 (2014) (Figure 3). The highest average yield gain of 25.4% was reached in 2018 when the combined occurrence of *U. nuda* and *P. graminea* was 472 plants per seed-untreated plot and when both pathogens were almost equally present. Although the occurrence of *P. graminea* in 2019 reached the highest value in the seven-year period (688 plants per seed-untreated plot), the yield gain sharply decreased to 5.3% (Figure 3). The level of infection with *U. nuda* in 2019 was only 15 plants per seed-untreated plot. In 2020, the combined occurrence of both pathogens reached 186 plants per seed-untreated plot, but *P. graminea* predominated over *U. nuda*, and the yield gain of 5.9% was in the range of that in 2014–2016.

Our results indicated that the contribution of fungicide treatment to yield gain depends not only on the overall level of pathogen pressure, but also on the predominance of one pathogen over the other. The highest contribution of fungicidal treatments to yield gain was achieved when the total number of plants infected with *U. nuda* and *P. graminea* per seed-untreated plot ranged from 400 to 500 and when *U. nuda* was the predominant pathogen, with more than 200 infected plants per seed-untreated plot (Figure 3). In these circumstances, the average yield gain of all applied fungicides was 17.4% (2017) to 25.4% (2018) (Figure 3).

**Figure 4:**
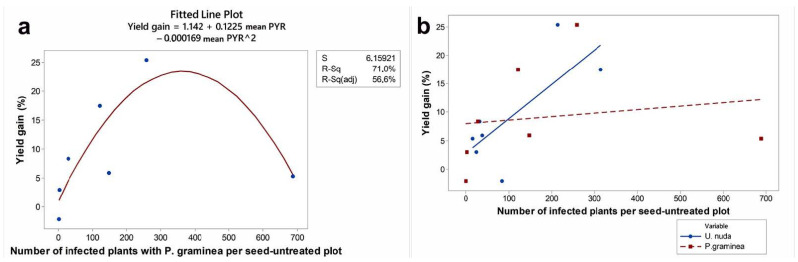
Regression analysis of the relationship between yield gain and pathogen pressure in the period 2014–2020: (**a**) Relationship between yield gain and total number of plants infected with *P. graminea* in the seed-untreated plot; and (**b**) relationship between yield gain and total number of plants infected with *U. nuda* in the seed-untreated plot.

**Figure 5:**
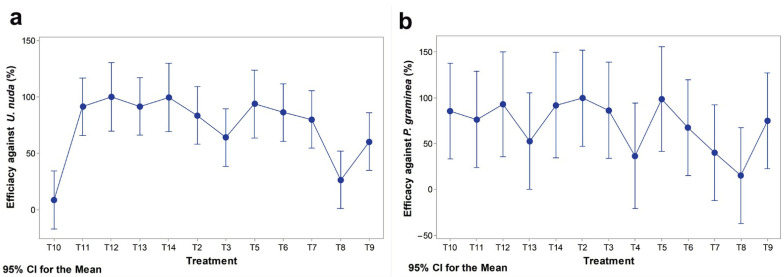
Fungicide efficacy against *U. nuda* (**a**) and *P. graminea* (**b**) in the period 2014–2020; T2—RAXIL S 040 FS; T3—VITAVAX 200 FF; T4—MANKOGAL S; T5—VIBRANCE DUO; T6—RANCONA TRIO; T7—CERTICOR 050 FS; T8—CELEST EXTRA 050 FS; T9—DIVIDEND EXTREME 115 FS (A12532C); T10—CELEST TOP 312.5 FS; T11—YUNTA QUATTRO; T12—VIAL TRUST FS; T13—LAMARDOR FS 400; T14—LAMARDOR FS 400 + GAUCHO 600 FS.

The exact relationship between yield gain after fungicide treatment and the pressure of a single pathogen could not be examined because of multiple seed-borne infections. However, there was an indication that the relationship between yield in seed-treated and seed-untreated plots was more uniformly related to the level of infection with *U. nuda* than with *P. graminea*. This was confirmed when polynomial regression was applied to yield gain, as the dependent variable and the total number of plants infected with *P. graminea* or *U. nuda* per seed-untreated plot as independent variables. The relationship between yield gain and the *P. graminea* pressure was significantly explained with quadratic regression (*p* = 0.037), contrary to linear (*p* = 0.73) and cubic (*p* = 0.65) ones (Figure 4a). Quadratic regression also fit the data well (*p* = 0.004) if yield gain was regressed with the occurrence of *P. graminea* in seed-treated plots. In contrast, the relationship between yield gain and the total number of plants infected with *U. nuda* per seed-untreated plot was better explained by linear regression (*p* = 0.05) than by quadratic (*p* = 0.73) and cubic regression (*p* = 0.06), giving an R^2^ of 56.1% (Figure 4b). The efficacy of individual fungicides against *P. graminea* was diverse in the seven-year period, resulting in a lack of significant differences among them (Figure 5b). Contrary to the efficacy of individual fungicide treatments against *P. graminea*, fungicide treatments against *U. nuda* differed significantly in the seven-year period (Figure 5a).

LAMARDOR + GAUCHO 600 FS (T14) had the highest and the most stable efficacy against *U. nuda* in the seven-year period together with VIAL TRUST (T12), which exceeded 99.5% (Table 5, Figure S3b). However, their contribution to yield gain exceeded 30% only in 2018. In the rest of the years, their contribution to yield gain was diverse and lower than 13.5% (Table S2, Figure S3a).

More interestingly, the contribution of triazole and benzimidazole-based fungicides to yield gain in 2017 was not significantly different from those having lower efficacy against *U. nuda,* such as the combination of fludioxonil and difenoconazole in CELEST TOP 312.5 FS and CELEST EXTRA 050FS. In addition, although CELEST TOP 312.5 FS (T10) had low average efficacy against *U. nuda* in the seven-year period (8.8%), its contribution to yield gain in 2018 (39.25%) was not significantly different from fungicides carrying more efficient active ingredients against *U. nuda* (Table S2). The efficacy of CELEST TOP 312.5 FS (T10) against *U. nuda* in 2018 was 44% (Figure S3b). The fungicides RAXIL-S 040 FS (T2) and VIBRANCE DUO (T5) had the highest efficacy against *P. graminea* over a seven-year period (>98%). However, their contribution to yield gain was the highest in 2017, when *U. nuda* predominated *P. graminea* (Figure S3a).

## 4. Discussion

Knowing that the combined effects of abiotic and biotic factors on yield have rarely been studied and are usually related to investigations in controlled conditions [18,22], this study examined potentials of regression models to explain yield loss in barley as a result of plant response to combinatorial stressors. Our results indicated that only combining abiotic and biotic predictor variables into the same regression model would give higher potentials for yield loss predictions. We also showed different contributions of pathogens to final yield achievements, and pointed out that more attention should be given to limits of infection with *U. nuda* and *P. graminea* that would cause yield loss in combination with other environmental factors.

The co-occurrence pattern of *U. nuda* and *P. graminea* was not uniform in the seven-year period. The most influential climatic factors on the occurrence of *U. nuda* in the seed-untreated plots were in accordance with previous reports indicating that the duration of floret opening affects spore entry and subsequent infection with *U. nuda* [10,23]. Woldemichael [24] reported that temperatures ranging from 15 to 22 °C promote longer flowering, allowing more time for teliospores of *U. nuda* to land on florets. Although it was reported that a relative humidity of 95% is the most conducive for teliospore germination and growth of *U. nuda* [25], the relative humidity at the time of flowering did not exceed 79% in the seven-year period. The significant influence of temperature in November on occurrence of *U. nuda* supports previous studies showing that the dynamics of early plant growth and the growth rate of rootles promote or initiate escape from infection with seed-borne pathogens [10].

Infection with *P. graminea* can take place from flowering time to the soft dough stage with conidia that are produced on the infected leaves. It was reported that more infection with *P. graminea* occurs when the relative humidity is near 100% at temperatures from 15 to 25 °C, with an optimum at approximately 22 °C. The maximum infection with *P. graminea* occurred in 2019, when the temperature at flowering time in the previous growing season reached a maximum value in the seven-year period (20.5 °C). Average temperature and total rainfall in the 10-day period at the time of sowing were distinguished as factors affecting the occurrence of only *P. graminea*. This is in accordance with reports of previous studies indicating that soil temperature during seedling emergence is critical for infection with *P. graminea* [26]. Tekauz et al. [26] reported that infection with *P. graminea* was promoted with soil temperatures below 15 °C and reduced sharply near 20 °C and above. However, there are also reports that temperatures above 15 °C reduced infection, while soil temperatures below 12 °C promoted *P. graminea* infection in Mediterranean countries under winter sowing and in Nordic countries under spring sowing [27].

Many components involved in the regulatory network for plant responses to abiotic and biotic stressors may function antagonistically [28,29,30], and in field conditions, it could jeopardize the prognosis of yield outcomes. In this study, the best subset regression indicated that using only biotic factors as an explanatory variable for yield loss would give a lower R^2^ (47.9%), lower R^2^ pred (39.3%) and much higher Mallows’ Cp (30.7) than using it in combination with abiotic factors. These results are in accordance with results reported by Jevtić et al. [31], who noted that estimation of the combined effect of obligate pathogens and climatic conditions on yield loss of susceptible wheat varieties would give regression models with higher R^2^ and R^2^ (pred). Temperature in the phenological phase of leaf development and flowering time together with the combined occurrence of *U. nuda* and *P. graminea* had the strongest impact on yield loss in the Krajišnik variety.

The influence of heat stress on yield achievements was reported in previous investigations [32,33], but there are no reports on its combined effect with *U. nuda* and *P. graminea*. In this study, flowering time contributed to yield loss together with loose smut and barley stripe. The joint occurrence of *U. nuda* and *P. graminea* in 2018 (472 infected plants per seed-untreated plot) and 2017 (436 infected plants per seed-untreated plot) was not significantly different, but yield loss in 2018 (29.5%) exceeded that in 2017 (19.7%). Greater yield loss in 2018 was affected by temperatures in flowering time that were almost 8 °C higher in 2018 than in 2017. The average temperature in the last 10 days of April 2018 (20.5 °C) and the first 10 days in May (20.9 °C) exceeded those in April (11.8 °C) and May (14.7 °C) 2017. The same was true for the grain-filling stage since the average temperature in May 2018 (20.4 °C) exceeded that in 2017 (17.6 °C) and the seven-year average (13.7 °C). Heat stress during different phenological phases affects different components of yield. In the period of preanthesis and anthesis, heat stress strongly limits grain number by influencing pollen germination, pollen tube growth and ovary development [34]. High temperature during the grain-filling period accelerates crop senescence, affecting shorter grain-filling stages and consequently grain weight [35]. The lower hardiness of plants infected with *U. nuda* and *P. graminea* in the seed-untreated plots additionally contributed to the negative effect of high temperatures on flowering time on yield.

In this study, neither fungicidal treatment had a stable contribution to yield gain in the seven-year period. It highlighted the complexity of factors influencing the contribution of fungicidal treatments to yield gain. Although triazole- (tebuconazole, prothioconazole) and benzimidazole- (tiabendazole) based fungicides showed high efficacy against *U. nuda* in a seven-year period, their contribution to yield gain was not straightforward. The difference in yield responses to fungicidal treatments could be due to differences in climatic factors influencing plant growth and seed formation. As shown above, climatic factors in the flowering and seed formation phenological stages were more conducive in 2017 than in 2018, resulting in fewer differences in yield achievements in the seed-treated and non-seed treated plots. Carboxin was reported as a popular systemic fungicide worldwide for controlling loose smut and reducing disease to low levels [36,37]. However, this study showed lower efficacy against *U. nuda* when compared with triazole-based fungicides. The occurrence of carboxin-resistant strains of *U. nuda* has been reported in France and Italy [38], but in this study, although the overall efficacy of fungicides containing carboxin was lower than that of triazole-based fungicides, its efficacy was dependent on year. In this study, both groups of fungicides contributed to yield gain without significant differences in most of the years.

Although chemical treatments have been proven to be a powerful disease-control tool, the question of their contribution to grain yield remains unanswered [39]. The contribution of fungicide treatment to yield gain is usually reported in two- or three-year studies, which is a limited time span to investigate the effect of diverse environmental factors and changes in seed-borne pathogen prevalence on fungicide contribution to yield gain. A significant influence of environmental factors on fungicide contribution to yield gain was already reported in the management of wheat foliar diseases [40,41], but similar studies for barley pathogens are still missing. Teng [42] reported that there is a problem with models that rely solely on the quantification of visible disease symptoms without considering the variations in growing conditions that occur between seasons. Hitaj et al. [43] indicated that there is a high degree of uncertainty about the amount of regional variability and the use of certain active ingredients for pesticides applied as seed treatments. It also raises the question of their contribution to yield gain under diverse environmental factors. In this study, it was revealed that the relationship between fungicide efficacy against *U. nuda* and *P. graminea* and yield gain in a seven-year period was not straightforward and was highly influenced by fluctuations in climatic factors at the time of flowering, overall pathogen pressure and predominance of one pathogen over the other. This result indicated that more attention should be given to the combined effects of abiotic and biotic factors to explain and predict yield achievements and ensure sustainable barley production.

## 5. Conclusions

The co-occurrence pattern of *U. nuda* and *P. graminea* was not uniform in the seven-year period. Shifts in the predominance of one pathogen over the other were shown, and differences in their effect on yield were indicated. The contribution of fungicide treatment to yield gain was more linearly related to plant infection with *U. nuda* than with *P. graminea*.

This study indicated that the prediction of barley yield losses should not be based only on the data of the number of infected plants with loose smut and barley stripe. Only combining abiotic and biotic predictor variables into the same regression model would give higher R^2^ values, lower Mallows’ Cp and higher potentials for yield loss predictions.

Temperature in the phenological phase of leaf development and flowering time together with the combined occurrence of *U. nuda* and *P. graminea* had the strongest impact on yield loss in the Krajišnik variety. This indicated that factors influencing yield loss should be analyzed as complex environmental systems.

Neither fungicidal treatment had a stable contribution to yield gain in the seven-year period. Extreme fluctuations in climatic factors at the time of flowering, overall pathogen pressure and predominance of one pathogen over the other were determined to be influencing factors on the contribution of fungicidal treatments to yield gain.

## Figures and Tables

**Table 1:** The sowing, flowering and harvesting dates of variety Krajišnik in a seven-year period.

Year	Heading Date	Flowering Date (Period When Infection Occurred) *	Sowing Date	Year	Harvest Date
2013	30 April	3 May	23 October	2014	19 June
2014	23 April	26 April	4 November	2015	16 June
2015	29 April	2 May	30 October	2016	23 June
2016	28 April	1 May	25 October	2017	17 June
2017	28 April	1 May	19 October	2018	7 June
2018	25 April	28 April	23 October	2019	28 June

* In statistical analysis on influencing factors on occurrence of *U. nuda* and *P. graminea*, climatic factors at the time of flowering were used from the previous growing season when infection occurred.

**Table 2:** Fungicide treatments in research and development trials on fungicide efficacy against *U. nuda* and *P. graminea*.

	Treatments	Dosage Rate (Amount/1 kg of Seed)
T1	Seed-untreated plot (Control)	
T2	RAXIL S 040 FS (20 g/L tebuconazole + 20 g/L triazoxide)	1 mL
T3	VITAVAX 200 FF (200 g/L carboxin + 200 g/L thiram)	3 mL
T4	MANKOGAL S (600 g/kg mancozeb)	2 g
T5	VIBRANCE DUO (25 g/L fludioxonil +25 g/L sedaxane)	2 mL
T6	RANCONA TRIO (5 g/L ipconazole + 13.3 g/L metalaxyl + 133 g/L carboxin)	1 mL
T7	CERTICOR 050 FS (30 g/L tebuconazole + 20 g/L metalaxyl -M)	1 mL
T8	CELEST EXTRA 050 FS (25 g/L fludioxonil + 25 g/L difenoconazole)	2 mL
T9	DIVIDEND EXTREME 115 FS (A12532C) (7.73% difenoconazole + 1.93% metalaxyl -M)	1.5 mL
T10	CELEST TOP 312.5 FS (262.5 g/L thiamethoxam + 25 g/L difenoconazole + 25 g/L fludioxonil)	1.5 mL
T11	YUNTA QUATTRO (6.7 g/L tebuconazole +33.3 g/L prothioconazole + 166.7 g/L imidacloprid +166.7 g/L clothianidin	1.8 mL
T12	VIAL TRUST FS (60 g/L tebuconazole+ 80 g/L thiabendazole)	4 mL
T13	LAMARDOR FS 400 (150 g/L tebuconazole + 250 g/L prothioconazole)	0.2 mL
T14	LAMARDOR FS 400 (150 g/L tebuconazole + 250 g/L prothioconazole) + GAUCHO 600 FS (600 g/L imidacloprid)	0.2 + 1.7 mL

**Table 3:** Regression analysis of the most influential factors on the occurrence of *U. nuda* and *P. graminea* in the period 2014–2020.

Source	DF	Adj SS	Adj MS	F Value	*p* Value
			*U. nuda*		
Regression	4	321,150	80,288	72.20	0.000
Rainfall (flowering time in previous season)	1	64,158	64,158	57.70	0.000
Temperature (flowering time in previous season)	1	22,899	22,899	20.59	0.000
Humidity (flowering time in previous season)	1	10,438	10,438	9.39	0.006
Temperature in November	1	187,980	187,980	169.04	0.000
Error	23	25,577	1112		
Total	27	346,727			
			*P. graminea*		
Regression	5	1,418,983	283,797	103.80	0.000
Rainfall (flowering time in previous growing season)	1	193,806	193,806	7089	0.000
Temperature (flowering time in previous season)	1	713,053	713,053	260.81	0.000
Humidity (flowering time in previous season)	1	350,595	350,595	128.23	0.000
Temperature (sowing time)	1	402,556	402,556	147.24	0.000
Rainfall (sowing time)	1	167,962	167,962	61.43	0.000
Error	22	60,149	2734		
Total	27	1,479,131			

**Table 4:** Best subsets regression analysis of the most influencing factors on yield loss of variety Krajišnik in the period 2014–2020.

Number of Predictors	R^2^	R^2^_pred_	Mallows’ Cp	S	Joined Occurrence of *U. nuda* and *P. graminea*	T in November (°C)	T in February (°C)	T in April (°C)
1	47.9	39.3	30.7	5.3481	X			
1	47.1	38.9	31.5	5.3861				X
2	70.8	63.0	9.1	4.0848	X			X
2	64.5	57.1	15.6	4.5034		X		X
3	77.7	71.2	4.0	3.6455	X	X		X
3	74.0	65.2	7.8	3.9355	X		X	X
4	78.6	70.7	5.0	3.6485	X	X	X	X

**Table 5:** Fungicide efficacy against *U. nuda* and *P. graminea* in 2014–2020.

Fungicide Treatment	Mean	Minimum	Maximum
	*U. nuda*		
T2 RAXIL S 040 FS (20 g/L tebuconazole +20 g/L triazoxide)	83.5 ^A B^	−4.1	100.0
T3 VITAVAX 200 FF (200 g/L carboxin + 200 g/L thiram)	64.1 ^A B C^	−12.4	96.1
T4 MANKOGAL S (600 g/kg mancozeb)	N/A *		
T5 VIBRANCE DUO (25 g/L fludioxonil + 25 g/L sedaxane)	93.80 ^A^	84.21	100.00
T6 RANCONA TRIO (5 g/L ipconazole + 13.3 g/L metalaxyl + 133 g/L carboxin)	86.29 ^A B^	60.82	100,00
T7 CERTICOR 050 FS (30 g/L tebuconazole + 20 g/L metalaxyl -M)	80.1 ^A B^	−27.8	100.0
T8 CELEST EXTRA 050 FS (25 g/L fludioxonil + 25 g/L difenoconazole)	26.7 ^B C^	−65.8	83.9
T9 DIVIDEND EXTREME 115 FS (A12532C) (7.73% difenoconazole + 1.93% metalaxyl -M)	60.4 ^A B C^	−8.6	94.1
T10 CELEST TOP 312.5 FS (262.5 g/L thiamethoxam + 25 g/L difenoconazole + 25 g/L fludioxonil)	8.8 ^C^	−59.7	70.6
T11 YUNTA QUATTRO (6.7 g/L tebuconazole + 33.3 g/L prothioconazole + 166.7 g/L imidacloprid + 166.7 g/L clothianidin	91.38 ^A^	69.74	100.00
T12 VIAL TRUST FS (60 g/L tebuconazole + 80 g/L thiabendazole)	100.00 ^A^	100.00	100.00
T13 LAMARDOR FS 400 (150 g/L tebuconazole + 250 g/L prothioconazole)	91.69 ^A^	43.30	100.00
T14 LAMARDOR FS 400 (150 g/L tebuconazole + 250 g/L prothioconazole) + GAUCHO 600 FS (600 g/L imidacloprid)	99.605 ^A^	98.026	100.000
	*P. graminea*		
T2 RAXIL S 040 FS (20 g/L tebuconazole + 20 g/L triazoxide)	99.488 ^A^	98.548	100.000
T3 VITAVAX 200 FF (200 g/L carboxin + 200 g/L thiram)	86.41 ^A^	53.18	100.00
T4 MANKOGAL S (600 g/kg mancozeb)	36.6 ^A^	−39.0	85.0
T5 VIBRANCE DUO (25 g/L fludioxonil + 25 g/L sedaxane)	98.645 ^A^	94.869	100.000
T6 RANCONA TRIO (5 g/L ipconazole + 13.3 g/L metalaxyl + 133 g/L carboxin)	67.38 ^A^	50.00	86.99
T7 CERTICOR 050 FS (30 g/L tebuconazole + 20 g/L metalaxyl -M)	39.9 ^A^	−80.0	87.2
T8 CELEST EXTRA 050 FS (25 g/L fludioxonil + 25 g/L difenoconazole)	15.1 ^A^	−370.0	100.0
T9 DIVIDEND EXTREME 115 FS (A12532C) (7.73% difenoconazole + 1.93% metalaxyl -M)	74.7 ^A^	−10.0	94.9
T10 CELEST TOP 312.5 FS (262.5 g/L thiamethoxam + 25 g/L difenoconazole + 25 g/L fludioxonil)	85.57 ^A^	60.00	98.62
T11 YUNTA QUATTRO (6.7 g/L tebuconazole + 33.3 g/L prothioconazole + 166.7 g/L imidacloprid +166.7 g/L clothianidin	76.45 ^A^	60.00	94.43
T12 VIAL TRUST FS (60 g/L tebuconazole + 80 g/L thiabendazole)	93.13 ^A^	80.90	98.23
T13 LAMARDOR FS 400 (150 g/L tebuconazole + 250 g/L prothioconazole)	52.8 ^A^	−100.0	96.5
T14 LAMARDOR FS 400 (150 g/L tebuconazole + 250 g/L prothioconazole) + GAUCHO 600 FS (600 g/L imidacloprid)	91.96 ^A^	83.06	99.16

* Not applicable. Means that do not share a letter are significantly different.

## Data Availability

Data are reported within the article or supplementary material.

## Supplementary Materials

The following supporting information can be downloaded at: https://www.mdpi.com/article/10.3390/jof8050542/s1. Figure S1. Assessments of *U. nuda* and *P. graminea* on locality Rimski šančevi in 2017: (a) Assessment of *U. nuda*; (b) Assessment of *P. graminea*. Figure S2: Average occurrence of *U. nuda* and *P. graminea* in seed-untreated plot in the period 2014–2020. Figure S3: (a) Contribution of fungicide treatments to yield gain in seven-year period; (b) Fungicide efficacy against *U. nuda* in seven-year period; (c) Fungicide efficacy against *P. graminea* in seven-year period. T1-Control; T2- RAXIL S 040 FS; T3- VITAVAX 200 FF; T4-MANKOGAL S; T5-VIBRANCE DUO; T6- RANCONA TRIO; T7-CERTICOR 050 FS; T8-CELEST EXTRA 050 FS; T9-DIVIDEND EXTREME 115 FS (A12532C); T10-CELEST TOP 312.5 FS; T1-YUNTA QUATTRO; T12- VIAL TRUST FS; T13- LAMARDOR FS 400; T14-LAMARDOR FS 400 + GAUCHO 600 FS. Table S1: Climatic factors at the time of flowering, sowing, emergence and leaf development that influenced the occurrence of *U. nuda* and *P. graminea* in a seven-year period. Table S2: Yield gain in seed-treated plots in the period 2014–2020.
