# Supplementary material for: Co-Occurrence Patterns of Ustilago nuda and Pyrenophora graminea and Fungicide Contribution to Yield Gain in Barley under Fluctuating Climatic Conditions in Serbia
Source: J Fungi (Basel). 2022 May 23;8(5):542. doi: 10.3390/jof8050542 (PMC9148124; doi:10.3390/jof8050542)
Supplement: Supplementary file 1 [file jof-08-00542-s001.zip › Supplementary Figure S1.pdf]

Co-Occurrence Patterns of *Ustilago nuda* and *Pyrenophora graminea* and Fungicide Contribution to Yield Gain in Barley under Fluctuating Climatic Conditions in Serbia

Radivoje Jevtić *, Vesna Župunski, Mirjana Lalošević, Ljiljana Brbaklić and Branka Orbović

Institute of Field and Vegetable Crops, 21000 Novi Sad, Serbia; vesna.zupunski@ifvcns.ns.ac.rs (V.Ž.); mirjana.lalosevic@ifvcns.ns.ac.rs (M.L.); ljiljana.brbaklic@ifvcns.ns.ac.rs (L.B.); branka.orbovic@ifvcns.ns.ac.rs (B.O.)

* Correspondence: radivoje.jevtic@ifvcns.ns.ac.rs; Tel.: +381-21-4898207

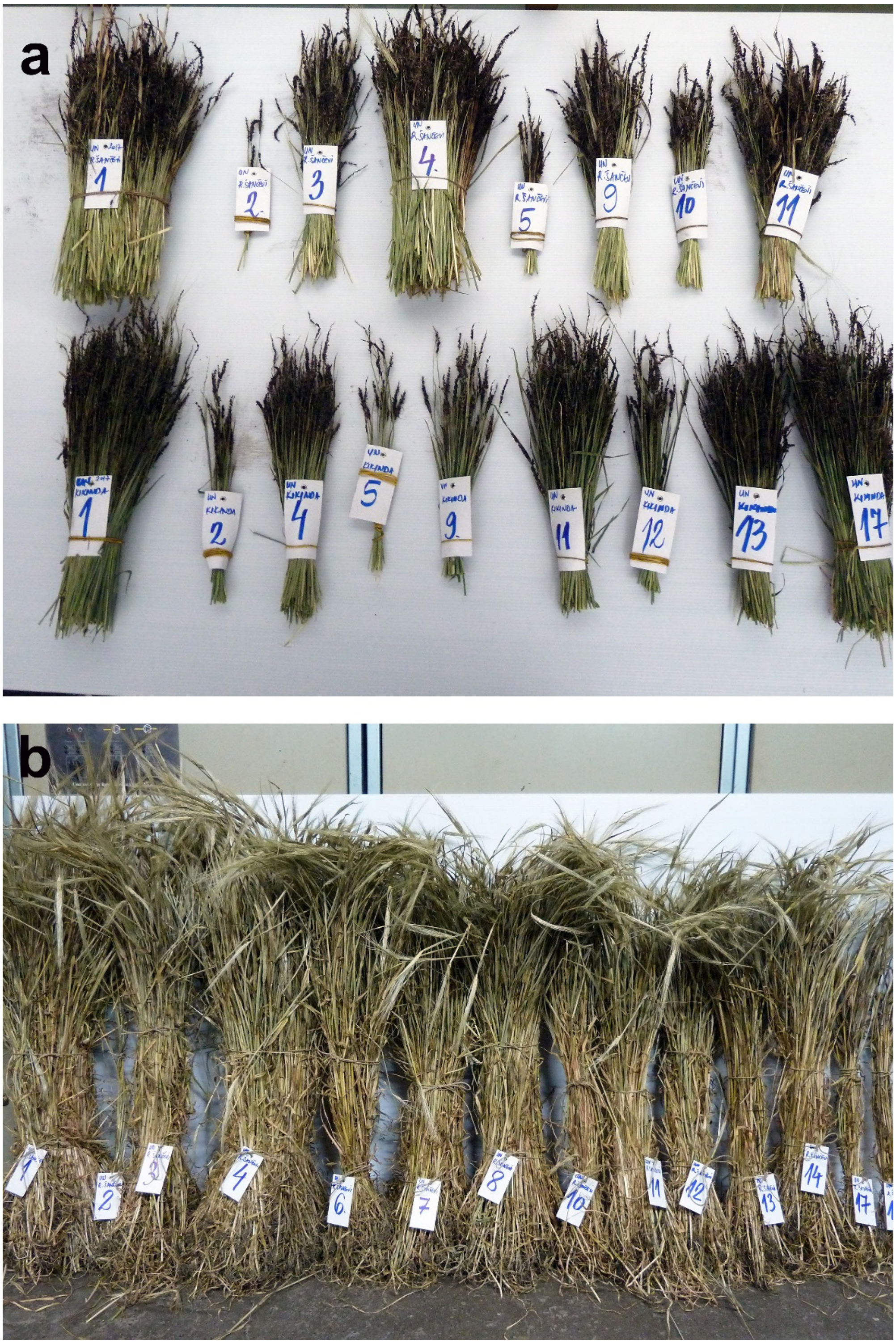

Figure S1. Assessments of *U. nuda* and *P.graminea* on locality Rimski šančevi in 2017: (**a**) Assessment of *U. nuda;* (**b**) Assessment of *P. graminea*
