# Supplementary material for: Co-Occurrence Patterns of Ustilago nuda and Pyrenophora graminea and Fungicide Contribution to Yield Gain in Barley under Fluctuating Climatic Conditions in Serbia
Source: J Fungi (Basel). 2022 May 23;8(5):542. doi: 10.3390/jof8050542 (PMC9148124; doi:10.3390/jof8050542)
Supplement: Supplementary file 1 [file jof-08-00542-s001.zip › Supplementary Figure S3.pdf]

Co-Occurrence Patterns of *Ustilago nuda* and *Pyrenophora graminea* and Fungicide Contribution to Yield Gain in Barley under Fluctuating Climatic Conditions in Serbia

Radivoje Jevtić *, Vesna Župunski, Mirjana Lalošević, Ljiljana Brbaklić and Branka Orbović

Institute of Field and Vegetable Crops, 21000 Novi Sad, Serbia; vesna.zupunski@ifvcns.ns.ac.rs (V.Ž.); mirjana.lalosevic@ifvcns.ns.ac.rs (M.L.); ljiljana.brbaklic@ifvcns.ns.ac.rs (L.B.); branka.orbovic@ifvcns.ns.ac.rs (B.O.)

* Correspondence: radivoje.jevtic@ifvcns.ns.ac.rs; Tel.: +381-21-4898207

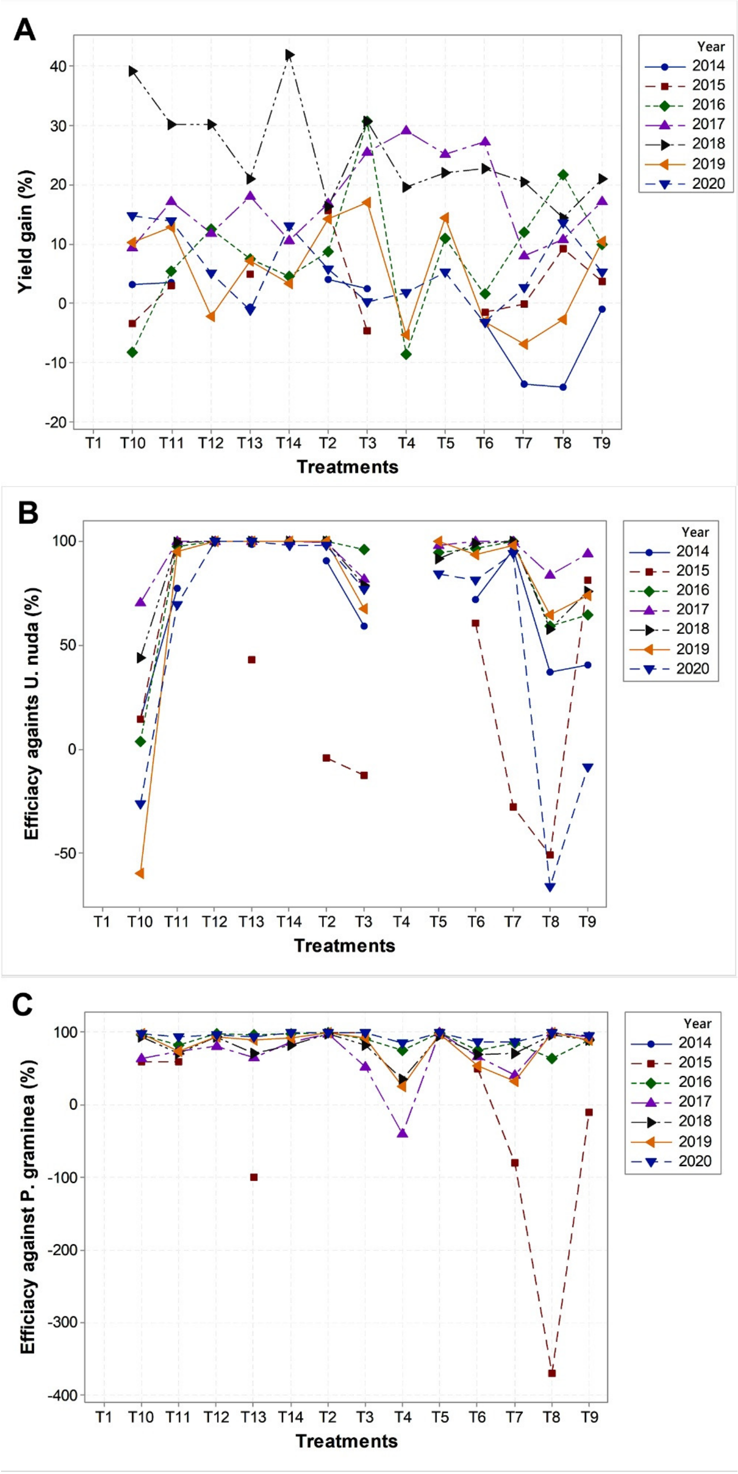

Figure S3. (**A**) Contribution of fungicide treatments to yield gain in seven-year period; (**B**) Fungicide efficacy against *U. nuda* in seven-year period; (**C**) Fungicide efficacy against *P.graminea* in seven-year period. T1- Control; T2- RAXIL S 040 FS; T3- VITAVAX 200 FF; T4-MANKOGAL S; T5-VIBRANCE DUO; T6- RANCONA TRIO; T7-CERTICOR 050 FS; T8-CELEST EXTRA 050 FS; T9-DIVIDEND EXTREME 115 FS (A12532C); T10- CELEST TOP 312.5 FS; T1-YUNTA QUATTRO; T12- VIAL TRUST FS; T13- LAMARDOR FS 400; T14- LAMARDOR FS 400 + GAUCHO 600 FS .
