# Supplementary material for: Co-Occurrence Patterns of Ustilago nuda and Pyrenophora graminea and Fungicide Contribution to Yield Gain in Barley under Fluctuating Climatic Conditions in Serbia
Source: J Fungi (Basel). 2022 May 23;8(5):542. doi: 10.3390/jof8050542 (PMC9148124; doi:10.3390/jof8050542)
Supplement: Supplementary file 1 [file jof-08-00542-s001.zip › Supplementary Table S1.pdf]

Co-Occurrence Patterns of *Ustilago nuda* and *Pyrenophora graminea* and Fungicide Contribution to Yield Gain in Barley under Fluctuating Climatic Conditions in Serbia

Radivoje Jevtić *, Vesna Župunski, Mirjana Lalošević, Ljiljana Brbaklić and Branka Orbović

Institute of Field and Vegetable Crops, 21000 Novi Sad, Serbia; vesna.zupunski@ifvcns.ns.ac.rs (V.Ž.); mirjana.lalosevic@ifvcns.ns.ac.rs (M.L.); ljiljana.brbaklic@ifvcns.ns.ac.rs (L.B.); branka.orbovic@ifvcns.ns.ac.rs (B.O.)

* Correspondence: radivoje.jevtic@ifvcns.ns.ac.rs; Tel.: +381-21-4898207

Table S1. Climatic factors at the time of flowering, sowing, emergence and leaf development that influenced the occurrence of *U. nuda* and *P. graminea* in a seven-year period

Year	Total rainfall in 10-day period in the time of flowering (mm)*	Average T in 10-day period in the time of flowering (°C) *	Average humidity in 10-day period in the time of flowering (%) *	Total rainfall in 10-day period in the time of sowing (mm) *	Average soil T in 10-day period in the time of sowing (°C) *	T in November
2013	48,4	20,1	67	0	15	8.3
2014	19,5	15,7	76	0	8,5	8.5
2015	7,8	19	64	0,2	9	7.7
2016	27,1	14,3	79	20,8	11,5	6.3
2017	28,7	14,7	79	0,7	10,5	7.0
2018	3,8	20,5	54	3,1	14	7.9
2019	78,7	14,3	79	7,5	13,5	11.2
Average	**30.5**	**16.9**	**71.1**	**4.6**	**11.7**	**8.1**

* Climatic factors at the time of flowering and sowing included the 10-day period in the first, middle or last part of the month depending on the starting date of the phenological phase. Disease assessments were associated with climatic factors at the time of flowering from the previous growing season when infection occurred.
