# Supplementary material for: Co-Occurrence Patterns of Ustilago nuda and Pyrenophora graminea and Fungicide Contribution to Yield Gain in Barley under Fluctuating Climatic Conditions in Serbia
Source: J Fungi (Basel). 2022 May 23;8(5):542. doi: 10.3390/jof8050542 (PMC9148124; doi:10.3390/jof8050542)
Supplement: Supplementary file 1 [file jof-08-00542-s001.zip › Supplementary Table S2.pdf]

Co-Occurrence Patterns of *Ustilago nuda* and *Pyrenophora graminea* and Fungicide Contribution to Yield Gain in Barley under Fluctuating Climatic Conditions in Serbia

Radivoje Jevtić *, Vesna Župunski, Mirjana Lalošević, Ljiljana Brbaklić and Branka Orbović

Institute of Field and Vegetable Crops, 21000 Novi Sad, Serbia; vesna.zupunski@ifvcns.ns.ac.rs (V.Ž.); mirjana.lalosevic@ifvcns.ns.ac.rs (M.L.); ljiljana.brbaklic@ifvcns.ns.ac.rs (L.B.); branka.orbovic@ifvcns.ns.ac.rs (B.O.)

* Correspondence: radivoje.jevtic@ifvcns.ns.ac.rs; Tel.: +381-21-4898207

Table S2. Yield gain in seed-treated plots in the period 2014-2020.

	Fungicide Treatment	2014	2015	2016	2017	2018	2019	2020
				Average yield gain				
T2	RAXIL S 040 FS (20 g/l tebuconazole +20 g/l triazoxide)	4.07 ^{A}	15.64 ^{A}	8.76 ^{ABC}	16.88 ^{ABC}	16.35 ^{AB}	14.19 ^{AB}	5.78 ^{ABC}
T3	VITAVAX 200 FF (200 g/l carboxin+200 g/l thiram)	2.54 ^{A}	-4.57 ^{B}	30.71 ^{A}	25.54 ^{ABC}	**30.68** ^{AB}	16.96 ^{A}	0.25 ^{ABC}
T4	MANKOGAL S (600 g/kg mancozeb)	*	*	-8.63 ^{C}	**29.10** ^{A}	19.58 ^{AB}	-5.37 ^{CD}	1.73 ^{ABC}
T5	VIBRANCE DUO (25 g/l fludioxonil+25 g/l sedaxane)	*	*	11.05 ^{ABC}	25.16 ^{ABC}	22.13 ^{AB}	14.42 ^{AB}	5.19 ^{ABC}
T6	RANCONA TRIO (5 g/l ipconazole+13.3 g/l metalaxyl+133 g/l carboxin)	-3.08 ^{A}	-1.58 ^{B}	1.54 ^{BC}	27.22 ^{AB}	22.82 ^{AB}	-3.11 ^{BCD}	-3.308 ^{C}
T7	CERTICOR 050 FS (30 g/l tebuconazole+20 g/l metalaxyl -M)	-13.60 ^{A}	-0.22 ^{AB}	12.09 ^{ABC}	7.94 ^{C}	20.45 ^{AB}	-6.93 ^{D}	2.70 ^{ABC}
T8	CELEST EXTRA 050 FS (25 g/l fludioxonil+25 g/l difenoconazole)	-14.25 ^{A}	9.30 ^{AB}	21.81 ^{AB}	**10.82** ^{BC}	14.38 ^{B}	-2.71 ^{BCD}	13.64 ^{AB}

T9	DIVIDEND EXTREME 115 FS (A12532C) (7.73% difenoconazole+1.93% metalaxyl -M)	-0.93 A	3.64 A B	9.87 A B C	17.17 A B C	20.97 A B	10.41 A B C D	5.31 A B C
T10	CELEST TOP 312,5 FS (262.5 g/l thiamethoxam+25 g/l difenoconazole+25 g/l fludioxonil)	3.138 A	-3.48 B	-8.33 C	**9.38** B C	**39.25** A B	10.20 A B C D	14.81 A
T11	YUNTA QUATTRO (6.7 g/l tebuconazole+33.3 g/l prothioconazole+166.7 g/l imidacloprid+166.7 g/l clothianidin	3.48 A	3.05 A B	5.34 A B C	17.27 A B C	**30.24** A B	12.93 A B C	13.89 A B
T12	VIAL TRUST FS (60 g/l tebuconazole+80 g/l thiabendazole)	*	*	12.59 A B C	11.88 A B C	**30.2** A B	-2.27 B C D	5.14 A B C
T13	LAMARDOR FS 400 (150 g/l tebuconazole+250 g/l prothioconazole)	-0.71 A	4.95 A B	7.43 A B C	18.04 A B C	**21.09** A B	7.22 A B C D	-1.20 B C
T14	LAMARDOR FS 400 (150 g/l tebuconazole+250 g/l prothioconazole)+ GAUCHO 600 FS (600 g/l imidacloprid)	*	*	4.53 B C	**10.53** B C	**41.94** A	3.27 A B C D	13.04 A B

* Not applicable. Means that do not share a letter are significantly different
